# Congenital Adrenal Hyperplasias Presenting in the Newborn and Young Infant

**DOI:** 10.3389/fped.2020.593315

**Published:** 2020-12-22

**Authors:** Antonio Balsamo, Federico Baronio, Rita Ortolano, Soara Menabo, Lilia Baldazzi, Valeria Di Natale, Sofia Vissani, Alessandra Cassio

**Affiliations:** ^1^Pediatric Endocrinology Unit, Department of Medical and Surgical Sciences, Endo-ERN Centre IT11, S.Orsola-Malpighi University Hospital, Bologna, Italy; ^2^Genetic Unit, Department of Medical and Surgical Sciences, Endo-ERN Centre IT11, S.Orsola-Malpighi University Hospital, Bologna, Italy

**Keywords:** newborn, 21-hydroxylase deficiency, 11-hydroxylase deficiency, 20–22-desmolase deficiency, StAR deficiency, P-450 oxydoreductase deficiency, 3-beta hydroxysteroid dehydrogenase deficiency, 17-hydroxylase/17-20 lyase deficiency

## Abstract

Congenital adrenal hyperplasia includes autosomal recessive conditions that affect the adrenal cortex steroidogenic enzymes (cholesterol side-chain cleavage enzyme; 3β-hydroxysteroid dehydrogenase; 17α-hydroxylase/17,20 lyase; P450 oxidoreductase; 21-hydroxylase; and 11β-hydroxylase) and proteins (steroidogenic acute regulatory protein). These are located within the three major pathways of the steroidogenic apparatus involved in the production of mineralocorticoids, glucocorticoids, and androgens. Many countries have introduced newborn screening program (NSP) based on 17-OH-progesterone (17-OHP) immunoassays on dried blood spots, which enable faster diagnosis and treatment of the most severe forms of 21-hydroxylase deficiency (21-OHD). However, in several others, the use of this diagnostic tool has not yet been implemented and clinical diagnosis remains challenging, especially for males. Furthermore, less severe classic forms of 21-OHD and other rarer types of CAHs are not identified by NSP. The aim of this mini review is to highlight both the main clinical characteristics and therapeutic options of these conditions, which may be useful for a differential diagnosis in the neonatal period, while contributing to the biochemical evolution taking place in the steroidogenic field. Currently, chromatographic techniques coupled with tandem mass spectrometry are gaining attention due to an increase in the reliability of the test results of NPS for detecting 21-OHD. Furthermore, the possibility of identifying CAH patients that are not affected by 21-OHD but presenting elevated levels of 17-OHP by NSP and the opportunity to include the recently investigated 11-oxygenated androgens in the steroid profiles are promising tools for a more precise diagnosis and monitoring of some of these conditions.

## Introduction

The most common and representative example of the congenital adrenal hyperplasia (CAH) group of disorders (≥90%) is the 21-hydroxylase deficiency (**CYP21A2-D**). Less frequent types of CAH are 11β-hydroxylase deficiency (**CYP11B1-D**, up to 8% cases), 17α-hydroxylase/17–20 lyase deficiency (**CYP17A1-D**), 3β-hydroxysteroid dehydrogenase deficiency (**HDS3B2-D**), P450 oxidoreductase deficiency (POR-D), P450 cytochrome side-chain cleavage deficiency (**CYP11A1-D**), and StAR deficiency (**StAR-D**). In CYP21A2-D and CYP11B1-D, only adrenal steroidogenesis is affected, whereas a defect in the other enzymes also involves gonadal steroid biosynthesis (1, 2) (Table 1).

**Table 1:** A summary of genetic, early clinical, biochemical features, and therapy of the CAH deficiencies presenting in the 1st year of life [modified by (1)].

			STAR PROT OMIM 201710	CYP11A1 OMIM 118485	HSD3B2 OMIM 201810	CYP17A1 OMIM 202110	CYP21A2 OMIM 201910	CYP11B1 OMIM 202010	POR OMIM 201750
Genetics	Gene		***StAR***	***CYP11A1***	***HSD3B2***	***CYP17A1***	***CYP21A2***	***CYP11B1***	***POR***
	Locus		Chr. 8p11.23; 7 exons	Chr. 15q24.1; 9 exons	Chr. 1p12; 4 exons	*Chr*. 10q24.32; 8 exons	Chr. 6p21.33; 10 exons	Chr. 8q24.3; 9 exons	Chr. 7q11.23; 17 exons
Clinical/ biochemical features at birth	MC	Renin	↑↑	↑↑	↑↑	↓	↑↑	↑↓	↓↑
		Na/K	↓/↑	↓/↑	↓/↑	↔	↓/↑	↔,↓/↔, ↑	↔
		BP	↓	↓	↓	↑ (no in partial defects)	↓	↑	↑
		Neonatal SW	+++	+++	+++	–	+++	–	–
	GC	Neonatal AI	+++	+++	+++	±	+++	+++	++
		Hypoglycemia	++	++	++	–	±	–	–
	Andr	Genitalia	46,XY DSD	46,XY DSD	46,XY DSD; 46,XX DSD (mild in 25% of cases)	46,XY DSD; absence of secondary sexual characteristics in both sexes	46,XX DSD	46,XX DSD	46, XY DSD; 46,XX DSD 75% of cases
Other features	Adrenal	Gland size	↑↑	↓↓	↔	↔	↑↑	↑↔	↔
Biochemical diagnostic markers	MC		↓↓	↓↓	Normal/↓	Serum: ↑ DOC Urine:↑ MC/ GC and ↑ androgens/ GC metabolites	↓/↔/↑^*^	Serum: ↑ S and DOC Urine: ↑ THS, THDOC	Normal
	GC		↓↓	↓↓	↓	Serum ↑ B	Serum: ↑ 21-DOF; urine:21-DOF (P'TONE)	Serum ↓ F	Normal
	Andr		↓↓	↓↓	Serum: ↑ stimulated ratio of Δ4 over Δ5 steroids; Urine: ↑ ratios DHEA/GC Metabolites and 5PT/GC Metabolite*	↓↓	Serum: ↑ 17-OHP ↑ 11-KΔ4A, 11-KT Urine: ↑17HP,PT Saliva: ↑11-KΔ4A and 11-KT	↑	Blood: mild ↑ 17-OHP; ↑ Pregn, Prog, 17-OHP Urine: combined impairment of diagnostic ratios for CYP17A1 and CYP21A2; ↑ of Pregn metabolites (PD)
Therapy	Hydrocortisone		+	+	+	+	+	+	±
	Fludrocortisone		+	+	+	–	+	±	–
	Mineralocorticoid receptor antagonists		–	–	–	±	–	+	–

MC, mineralocorticoids; GC, glucocorticoids; Andr, androgens; K, potassium; BP, blood pressure; SW, salt wasting; AI, adrenal insufficiency. *Cross reaction with high levels of other adrenal steroids. For steroid abbreviations, see the specific table.

* *It can be notoriously difficult to diagnose HSD3B2-D on urine sample alone due to the naturally high levels of 3βOH5ene steroids in neonates*.

### Steroid Acute Regulatory Protein deficiency—Lipoid CAH (StAR-D)

#### Epidemiology/Genetics

StAR-D is uncommon in most populations, but it is relatively more frequent in East Asian (3, 4), Arab (5), and Swiss (6) populations because of the occurrence of the p.Q258X, p.R182L/p.R182I, and p.L260P founder mutations, respectively. To date, ~85 pathogenic variants of the *StAR* gene have been reported (www.hgmd.cf.ac.uk) (Table 1).

#### Essential Biochemistry

**Figure 1:**
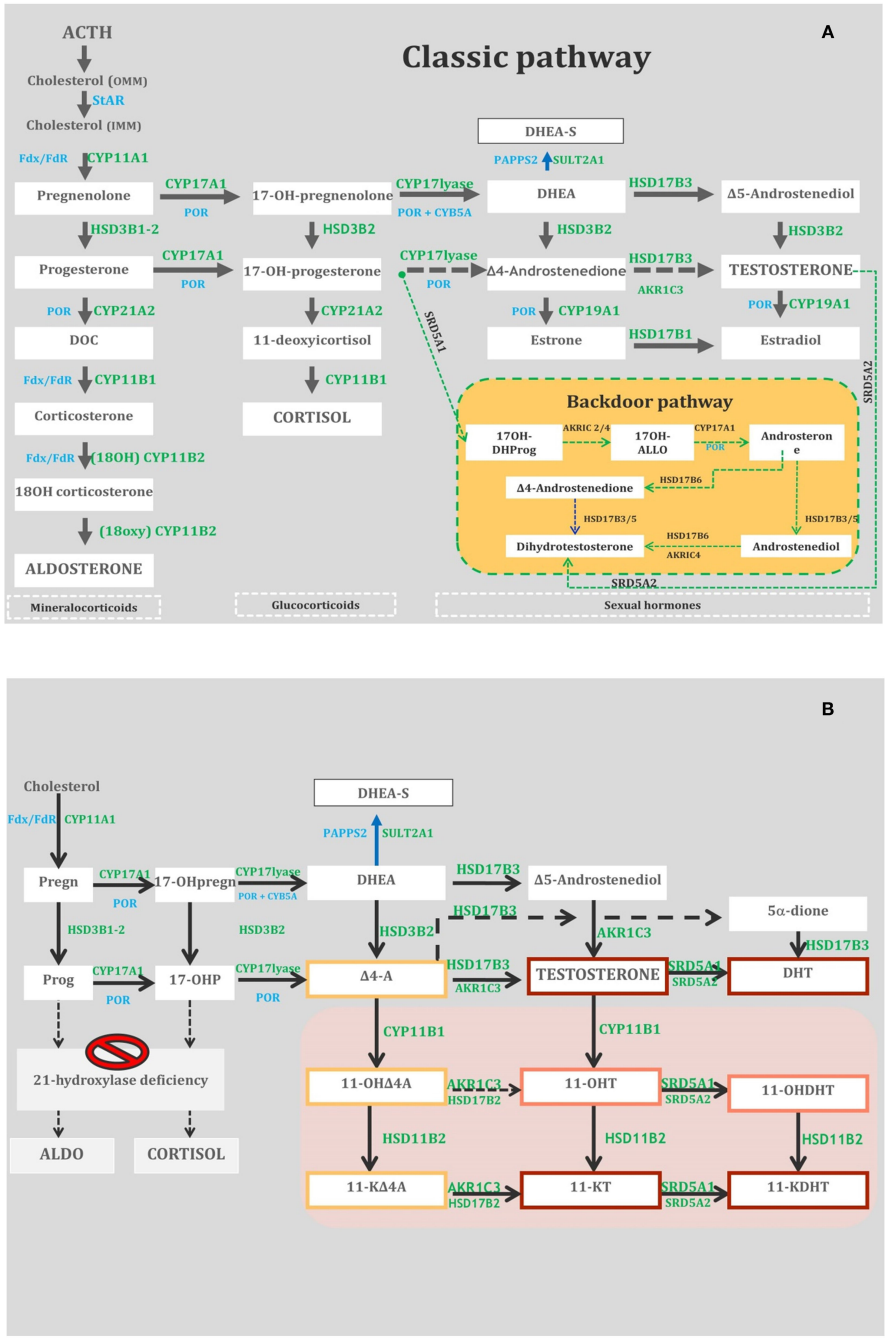
**(A)** Adrenal and gonadal steroidogenesis (Classic and Backdoor pathways). StAR, steroidogenic acute regulatory protein; OMM, outer microsomal membrane; IMM, inner microsomal membrane; DHEA/DHEA-S, Dehydroepiandrosterone/D-Sulfate; DOC, Deoxycorticosterone; 17OH-DHProg, 5-Pregnan-17-ol-3,20-dione (diol); 17OH-ALLO, 17OH-allopregnanolone; CYP11A1, cholesterol side-chain cleavage enzyme; CYP17A1, 17-hydroxylase/17, 20 lyase; SULT2A1, dehydroepiandrosterone (DHEA) sulfotransferase; POR, P450 oxidoreductase; CYP21A2, 21-hydroxylase; HSD3B2, 3-hydroxysteroid dehydrogenase; CYP11B1, 11-hydroxylase; CYP11B2, aldosterone synthase; HSD17B3, 17-hydroxysteroid dehydrogenase type 3; SRD5A1, 5-reductase type 1; SRD5A2, 5-reductase type 2; Fdx/ FdR, ferredoxin/ferredoxin reductase; CYB5A, cytochrome b5. **(B)** The metabolic pathways of classic and non-classic androgens. The gray box indicates 11-oxygenated C19 steroids. The red, orange, and yellow boxes depict steroids with strong, mild, and weak androgenic activities, respectively. HSD17B3, 17-hydroxysteroid dehydrogenase type 3; HSD3B2, 3-hydroxysteroid dehydrogenase type 2; AKR1C3, aldo-keto reductase family 1 member C3; CYP11B1, cytochrome P450 11B1; HSD11B1, 11-hydroxysteroid dehydrogenase type 1; HSD11B2, 11-hydroxysteroid dehydrogenase type 2; HSD17B2, 17-hydroxysteroid dehydrogenase type 2; SRD5A1, 5-reductase type 1; SRD5A2, 5-reductase type 2; Pregn, pregnenolone; 17-OHpregn, 17-OHpregnenolone; DHEA-S, dehydroepiandrosterone-sulfate; SULT2A1, sulfotransferase family 2A member 1; DHEA, dehydroepiandrosterone; Prog, progesterone; 17-OHP, 17-OH-progesterone; D4-A, androstenedione; 11-OHD4A, 11-hydroxyandrostenedione; 11-KD4A, 11-ketoandrostenedione; 11-OHT, 11-hydroxytestosterone; 11-KT, 11-ketotestosterone; 5-dione, 5-androstanedione; DHT, dihydrotestosterone; 11-OHDHT, 11-hydroxydihydrotestosterone; 11-KDHT, 11-ketodihydrotestosterone.

StAR is a fundamental actor in steroidogenesis, transferring cholesterol from the outer (OMM) to the inner mitochondrial membrane (IMM), where CYP11A1 can convert cholesterol to pregnenolone (Preg) (Figure 1A). The complex pathophysiology of StAR-D is explained by the “two-hit disease model” (5): the major part of steroidogenesis is StAR dependent, and its deficit, the first hit, activates the ACTH axis and *de novo* cholesterol biosynthesis; the consequent steroid underproduction due to the toxic effects of accumulating cholesterol follows as the second hit. The impairment of testicular steroidogenesis, which is active earlier than the ovarian one, is the first consequence of StAR-D with fetal androgen deficiency, causing undervirilization in 46,XY genetic newborns (7). Fetal adrenal androgen deficiency also leads to reduction of maternal estriol (E3) levels, prenatally measurable in a maternal urine sample (8). As placental steroidogenesis is not StAR dependent, Prog production is able to maintain pregnancy to term.

#### Clinical Presentation and Diagnosis

In its most severe form, the affected newborns cannot produce significant amounts of any steroid (9, 10). They show high ACTH levels, increased plasma renin activity, and engorged adrenal glands containing excessive amounts of cholesterol and its derivatives (5) (Table 1). Classic patients have severe salt loss within the 1st months of life and female external genitalia, irrespective of chromosomal sex (11). In 46,XY babies, Sertoli cells stay intact and the anti-Mullerian hormone (AMH) inhibits the development of Mullerian structures. The hydrosaline balance is controlled prenatally by the placenta, but mineralocorticoid (MC) deficiency emerges within 2–3 weeks due to progressive cellular destruction and some remaining StAR-independent MC biosynthesis. “As the ovary is steroidogenically quiescent until puberty, it is protected from cellular damage until steroidogenesis begins” (12, 13).

### P450 Cytochrome Side-Chain Cleavage Deficiency (CYP11A1-D)

#### Epidemiology/Genetics

CYP11A1-D is an even rarer defect than StAR-D, and it is caused by pathogenic variants of the *CYP11A1* gene (14). To date, 40 patients and 25 variations of *CYP11A1* have been reported. Almost all cases are homozygous or compound heterozygous (15). Autosomal dominant inheritance has also been proposed in a few cases (16, 17).

#### Essential Biochemistry/Pathophysiology

CYP11A1 catalyzes the conversion of cholesterol to Preg in three consecutive rate-limiting steps: 20α-hydroxylation, 22R-hydroxylation, and cleavage of the C20–C22 carbon side chain (18) (Figure 1A). CYP11A1-D determines defects in all three steroidogenic pathways: MC, glucocorticoid (GC) in the adrenals, and androgen (Andr) in the adrenals and gonads. Complete CYP11A1-D was considered incompatible with term pregnancies due to impaired placental progesterone and maternal estrone (E1) production; the reason why some cases survived pregnancy is still not completely clear (19–22). The expression of CYP11A1-D occurs early in fetal testes, causing defective gonadal steroidogenesis that dramatically impairs virilization of 46,XY fetuses (23).

#### Clinical Presentation and Diagnosis

In newborns, the most severe presentation is characterized by early adrenal insufficiency with salt wasting (SW), hypoglycemia, skin hyperpigmentation, and complete feminization of external genitalia, regardless of sex chromosomes. The 46,XY newborns show normal or hypoplastic derivatives of Wolffian duct and small testes, whereas derivatives of Mullerian duct are absent (11). Histology of the testes reveals immature tissue without germ cells (20). A phenotype with neonatal and transient adrenal insufficiency, life-threatening failure to thrive, and normal male external genitalia in 46,XY patients was reported in 2018 in three heterozygous related cases (17). One case of mid-shaft hypospadias and cryptorchidism at birth and another with penoscrotal hypospadias associated with late-onset adrenal insufficiency (9 and 2 years of age, respectively) were reported in 2009 (24) and 2012 (25). In newborns, blood tests showed severe hyponatremia, hyperkalemia, extremely elevated levels of ACTH, and renin activity with low or inappropriately normal levels of cortisol and aldosterone. Unlike most classic lipoid-CAH (26), adrenal glands are reduced in size in CYP11A1-D (27).

### 3β-Hydroxysteroid Dehydrogenase Deficiency (HSD3B2-D)

#### Epidemiology/Genetics

HSD3B2-D is a very rare form of CAH (estimated incidence of < 1/1,000,000 live births) (18, 28) caused by mutations in the *HSD3B2* gene (Table 1) that encode the 3β-HSD2 enzyme. It is involved in all three steroidogenic pathways: aldosterone, cortisol, and androgen precursors in the adrenals and testosterone (T) in the gonads (18). Loss-of-function mutations (<5% residual enzyme activity) predict the neonatal SW phenotype. Mutations causing >5% 3β-HSD2 activity lead to residual MC production without SW (29).

#### Essential Biochemistry/Pathophysiology

3β-HSD2 enzyme converts Δ5-3β-hydroxysteroids into corresponding Δ4-3-keto isomers, Preg to Prog, 17α-hydroxypregnenolone (17OHPreg) to 17α-hydroxyprogesterone (17OHP), dehydroepiandrosterone (DHEA) to Δ4-androstenedione (Δ4A), and androstenediol to T. In SW HSD3B2-D, glucocorticoid and mineralocorticoid are impaired causing hyponatremia, hyperkalemia, and elevated renin concentrations in both sexes. In females, 3β-HSD2 deficiency prevents the flooding of 17OHP and Δ4A to backdoor and 11-oxyandrogen production pathways (see CYP21A2-D) (Figures 1A,B); in males, T production is impaired during the critical period of sexual differentiation and dihydrotestosterone (DHT) production is subsequently reduced by classical and backdoor pathways (30).

#### Clinical Presentation and Diagnosis

Historically, the clinical presentation of HSD3B2-D at birth is described as the “classic form,” with or without SW, hypoglycemia, ambiguous genitalia, and hypogonadism in both sexes. Recent studies have shown that HSD3B2-D rarely causes ambiguous genitalia in females and thus the affected 46,XX newborns may present mild clitoromegaly only, whereas affected 46,XY newborns may present some degree of external genitalia undervirilization or isolated hypospadias, which need to be graded based on reliable tools (EGS) (31), (EMS) (32), (Prader) (33). The frequency of HSD3B2-D could be underestimated in females without SW and normal genitalia. However, in countries with NSP for 21-OHD, it is possible that newborns with HSD3B2-D may show false positivity for elevated levels of 17-OHP (34–37). The principal diagnostic test for HSD3B2-D is the serum measurement of 17-OHpreg, cortisol, Δ4A, 17-OHP, and DHEA (basal or post-ACTH stimulation) (28) with a predominance of Δ5 steroids (i.e., Preg, 17OHPreg, and DHEA) over Δ4 steroids (Prog, 17OHP, and Δ4A). Guran et al. (30) reported that high baseline 17OHpreg-to-cortisol ratio and low 11-oxyandrogen concentrations by LC/MSMS provide an unequivocal biochemical diagnosis of patients with HSD3B2-D. Although urinary steroid profiling is considered to be similarly accurate and less invasive for diagnosis (38), it can be notoriously difficult to diagnose HSD3B2-D on urine sample alone due to the naturally high levels of 3βOH5ene steroids in neonates.

### 17α-Hydroxylase/17,20 Lyase Deficiency (CYP17A1-D)

#### Epidemiology/Genetics

The incidence of CYP17A1-D is estimated to be about 1 in 50,000 (39). The disease prevalence is higher in certain countries such as the Netherlands (Friedlanders), Brazil, China, and Japan, where it is the second leading cause of CAH. This is attributable to loss-of-function mutations in the *CYP17A1* gene (40) (Table 1). Over 100 mutations in the *CYP17A1* gene are known, mostly resulting in complete loss of enzymatic activities of both 17-hydroxylase and 17–20 lyase (39). Researchers have also reported partial loss of enzymatic activity and loss of either hydroxylase or lyase activity alone (41).

#### Essential Biochemistry

CYP17A1 mediates three major transformations in cortisol and sex steroid biosynthesis. In particular, 17-hydroxylase mediates the synthesis of 17-Preg from Preg and 17OHP from Prog, whereas 17–20 lyase controls the production of DHEA from 17OHPreg. This latter step is of paramount importance as DHEA is the progenitor of steroid sex hormones (Figure 1A). The biochemical markers used to diagnose CYP17A1-D are shown in Table 1.

#### Clinical Presentation and Diagnosis

In 46,XX patients, external genitalia are normal on the neonatal exam as are the internal one to ultrasound (US). They may have no complaints before the typical age of puberty when the deficiency in sex hormones becomes apparent and they may develop hypertension or hypokalemia and high gonadotropin levels (hypergonadotropic hypogonadism).

In 46,XY patients, the presentation is typically under masculinization and can range from phenotypic female to ambiguous or small male genitalia (41). On physical examination, they may have a blind pouch instead of a vagina with a lack of internal female genitalia. The testes are undescended or located in the inguinal canal on imaging studies. Early diagnosis and treatment allow for the prevention of morbidity associated with hypertension, electrolyte abnormalities, and impairment of sexual development. As NSP identifies classic CYP21A2-D but does not detect CYP17A1-D, provider awareness and consideration of this condition are imperative for appropriate diagnosis.

### 21-Hydroxylase Deficiency (CYP21A2-D)

#### Epidemiology/Genetics

The most common form of CAH is represented by CYP21A2-D (90% cases). The severity of the enzymatic deficiency determines three clinical forms: SW (<1% enzyme activity), simple virilizing (SV; 1–2%), and non-classical (NC; 20–60%). The incidence of classic forms (SW and SV) ranges between 1 in 13,000 and 1 in 15,000 live births (42): in most populations, the frequency of heterozygous carriers is 1 in 60. CYP21A2-D is caused by mutations in the *CYP21A2* gene (6p21.3). Microconversions or apparent gene conversions that cause the transfer of an inactive pseudogene (CYP21P) to the functional gene are responsible for 95% of pathogenic variations (43). Rare patients with classic CAH (SW) show a “contiguous gene syndrome”, with CAH and Ehlers–Danlos Syndrome (EDS) features, which is called “CAH-X” (44).

#### Essential Biochemistry/Pathophysiology

In SW CYP21A2-D, GC, and MC production is severely impaired, whereas abnormal amounts of Andr are produced, stimulated by the increased levels of ACTH. 17OHP elevation represents the hallmark of the disease, and the large majority of classic CYP21A2-D patients show basal levels of 17OHP as >300 nmol/L (>10,000 ng/dL) (45). 17OHP is converted to T and 5α-DHT, two androgens with potent activity, by the so-called “front door” pathway and directly to DHT *via* an alternative pathway known as “the backdoor pathway” (46, 47) (Figure 1A). The latter could lead to hyperandrogenism less responsive to GC treatment (48). 11-Keto-testosterone (11KT) is derived from 11-hydroxylation of Δ4A and T by CYP11B1 and acts as a potent androgen with a fundamental role in the pathophysiology of classic CYP21A2-D (49) (Figure 1B). It could be utilized in the future as a more precise biochemical marker of the disease (measured by means of LC/MSMS) than DHEA, Δ4A, and T (49, 50).

#### Neonatal Presentation

All fetuses affected by classic CAH show varying degrees of genital virilization due to exposure to intrauterine androgen excess, so that any newborn with ambiguous genitalia or in extreme cases apparently male genitalia and non-palpable gonads (45) should be suspected of having SW CAH (2, 45). Patients with 46,XX very often show a vagina that opens into a common urogenital sinus with enlarged clitoris and normal cervix, uterus, and ovaries; 46,XY children may show macrogenitosomia and genital hyperpigmentation but are generally unrecognized at birth. Sodium loss and potassium retention occur in newborns with SW CAH, due to mineralocorticoid deficiency. This may be detected biochemically from 4 to 7 days of life, but takes longer to present clinically (2nd week to 1st month of life).

##### Newborn Screening

In several countries, NBS has been developed for early diagnosis of CYP21A2-D by measuring 17OHP blood levels on dried blood spots. NBS is fundamental in preventing SW crises in males and male sex assignment in affected females. The diagnosis of CYP21A2-D is made when 17OHP levels are above the cutoff levels that should be elaborated and adjusted for gestational age at each screening center (51). A second-tier test on the same blood sample by LC/MSMS multi-hormonal profile could improve the positive predictive value of the CAH screening (52) and be helpful in diagnosing other rarer forms of CAH (35, 53).

##### Prenatal Treatment

The prenatal diagnosis of affected CAH fetuses is usually made by chorionic villous sampling at 10–12 weeks of gestation or by amniocentesis at 15–16 weeks of gestation. Treatment in utero of potentially affected CAH patients is feasible by administering dexamethasone to the mother starting from the first weeks of pregnancy, with the aim of containing adrenal hyperandrogenism by reducing ACTH hypersecretion and avoiding genital masculinization in the CYP21A2-D female fetuses (2). However, it should still be considered an experimental therapy due to potential adverse effects on both unaffected children that need to be treated until diagnosis is achieved and their mothers (45). A non-invasive method using cell-free fetal DNA in maternal plasma (NIPD at 5 weeks of gestation) could allow selective treatment in affected females only (54) but is not routinely performed due to its complexity and associated cost.

### 11β-Hydroxylase Deficiency (CYP11B1-D)

#### Epidemiology/Genetics

11-OHD is among the most common causes of CAHs in the world, after 21-OHD, and accounts for about 5% of CAH patients with a European ancestry (55) and for about 15% of CAH patients in the Muslim and Jewish Middle Eastern populations (56). The classical form of 11-OHD has an estimated frequency of 1 in 200,000 live births (57). This is caused by mutations in the *CYP11B1* gene (Table 1). Approximately 130 mutations of the *CYP11B1* gene have been described so far.

#### Essential Biochemistry

In the normal adrenals, 11β-hydroxylase is expressed in the zona fasciculata and converts 11-deoxycortisol to cortisol in response to ACTH. 11β-hydroxylase and aldosterone synthase can convert DOC into corticosterone (B). 11-OHD disrupts the synthesis of cortisol with normal production of aldosterone (1). The key steroid used in diagnosis for the classic form is elevated 11-deoxycortisol basal levels (27). Serum B, DOC, and 17-OHP are also elevated, and elevated levels of the latter can cause CYP21A2-D misdiagnosis. The urinary metabolites, such as tetrahydro-cortisone, tetrahydro-11-deoxycorticosterone, and tetrahydro-11-deoxycortisol (2), are useful for diagnosis.

#### Clinical Presentation and Diagnosis

The classical form is characterized by excess androgen and hypertension. ACTH excess due to cortisol deficit causes overproduction of androgens and DOC: androgens lead to virilization similar to CYP21A2-D in affected female patients (46,XX DSD); excess of DOC causes low-renin hypertension (2). Hypertension might not be apparent during the neonatal period (in about one-third of patients) due to mineralocorticoid resistance, and some patients can present with salt loss during the neonatal period, especially after the start of the GC treatment (58).

### P450 Oxidoreductase Deficiency (POR-D)

#### Epidemiology/Genetics

POR-D was first described in 2004 (59) as a rare form of CAH. Currently, about 100 cases of POR-D have been reported worldwide with a broad clinical spectrum, and most occurring in neonates and children (60). Since 2004, some pathogenetic variants causing defective binding of co-factors and others causing altered interaction with partner proteins have been described (61). The homozygous null mutations appear to be lethal (62, 63).

#### Essential Biochemistry

POR is involved in the metabolism of drugs and steroid hormones because “all cytochrome P450 enzymes located in the endoplasmic reticulum get electrons for their catalytic activities from the co-factor” (61) NADPH through POR (64, 65). The main microsomal POR-dependent enzymes are involved in the biosynthesis of steroid hormones in both the adrenal cortex and gonads (CYP17A1, CYP19A1, CYP21A2, and CYP15A1), as well as in the metabolism of drugs and endogenous substrates (CYP3A4 and CYP2D6) in the liver (66–68). Several studies also suggest a possible role for POR in bone development and retinoic acid metabolism, which lead to skeletal anomalies (60).

#### Clinical Presentation and Diagnosis

POR-D was initially identified as a difference of sex development (DSD) with ambiguous genitalia similar to some cases of Antley–Bixler syndrome (ABS), a bone malformation syndrome due to the presence of mutations in FGFR2 (69). A recent review (60) meta-analyzed the phenotypic features in newborns with POR-D, with DSD at birth in 69% of patients (78% 46,XX and 60% 46,XY) (60). Maternal virilization during pregnancy, due to a defect in aromatase (CYP19A1) activity, is described in 21% of mothers, with the highest incidence (44.4%) when at least one of the mutations was R457H (60).

Skeletal malformations resembling ABS features were described in the 84% of PORD patients without mutations in *FGFR2* (60), such as midface hypoplasia (71%), large joint synostosis (69%), craniosynostosis (65%), hand and feet malformations (61%), and bowing of the femora (21%) (63). A latent form of adrenal insufficiency rarely becomes clinically evident in the neonatal period (70–72). “Due to a very complex effect on steroid metabolism, it is preferable to diagnose PORD by performing mass spectrometry analysis of urine and blood samples” (61). Hormonal analysis is characteristic of mild to moderate increase in 17OH progesterone levels (found through neonatal screening or biochemical analysis), normal baseline ACTH, and cortisol levels with an inadequate increase in cortisol production after ACTH stimulation, normal values for renin and aldosterone, and elevated values for progesterone, corticosterone, 18OH corticosterone, 11-deoxycorticosterone (DOC), 18OH DOC, and 21-deoxycortisol (59). Low E3 and increased metabolites of Preg in urine or amniotic fluid of the mother can be useful for prenatal diagnosis (73). However, definitive diagnosis of PORD needs to be done by genetic analysis of the *POR* gene.

## Therapeutic Approach

### Glucocorticoids (GCs)

Substitutive treatment with oral hydrocortisone (10–15 mg/m^2^/day, divided into three daily doses) is mandatory for all classic forms of CAH presenting during the neonatal period. In CYP21A2-D and CYP11B1-D, GC administration prevents further genital virilization. Higher doses (15–30 mg/m^2^/day, divided into 3 or 4 daily doses) are often indicated both initially, to slow down the excessive production of potentially unfavorable metabolites (21OHD), and subsequently, as neonates and young infants often require higher doses per surface area than older children or adults. Other forms of GC are not recommended due to possible ineffectiveness (cortisone acetate) or detrimental effects on child growth (prednisolone and dexamethasone). Alawi et al. (28) suggested administering hydrocortisone at slightly higher doses (12–18 mg/m^2^/day) in HSD2B3-D due to the greater difficulty in suppressing androgens.

We recommend educating parents and caregivers for adrenal crisis prevention and at least doubling the dose of GC (but not MC) for situations such as febrile illness (>38.5°C) and gastroenteritis with dehydration. Parenteral hydrocortisone administration (i.v. bolus of 12.5 mg in neonates and young infants, often with glucose and saline and administered within 3–10 min; bolus repetition every 4–6 h in the first 24 h or a continuous infusion of 100 mg/m^2^/day) is mandatory in cases of adrenal crisis with vomiting, major surgery accompanied by general anesthesia, and major trauma.

In patients <18 months of age, close monitoring in the first 3 months of life and every 3 months thereafter is recommended (45).

### Mineralocorticoids (MCs)

Fludrocortisone (0.05–0.2 mg, once or twice daily) together with sodium chloride administration during the first 6–12 months of life (5 mmol/kg/day, divided into 4–5 meals; 17 mmol or mEq = 1 g NaCl) aims to prevent adrenal crisis in StAR-D, CYP11A1-D, SW HSD2B3-D, and SW CYP21A2-D. Monitoring of K and BP levels is important for dose management.

### Other Medical Treatments

In undervirilized 46,XY newborns with StAR-D, CYP11A1-D, CYP17A1-D, or POR-D reared as male, sex steroid replacement (T or DHT) might be useful during minipuberty (74). In newborns with CYP11B1-D or CYP17A1-D, if blood pressure control is not achievable by glucocorticoids alone, then appropriate antihypertensives should also be administered. Sometimes, treatment with mineralocorticoid receptor antagonists may be necessary (75). In POR-D, the supplementation of sex steroids and glucocorticoids must be based on the steroid profile of the patient, considering the possibility of impaired drug metabolism. Skeletal malformations require orthopedic management. Potential therapeutic options include the introduction of external flavin (66) and treatment with cysteamine in case of arginine to cysteamine mutations (76).

### Surgical Treatment

A multidisciplinary team with competence in DSD management is recommended. In all pediatric patients with CAH, particularly minimally virilized girls (Prader I–II) and mildly undervirilized boys (EMS 7–11) (32), parents must be informed about surgical options, including delaying surgery and observation until the child is older.

Usually, the sex assignment in 46,XX newborns with CYP21A2-D or CYP11B1 is female, and genital surgery may be necessary, but the timing of the surgery remains controversial. In patients for whom early surgery is selected, vaginoplasty using urogenital mobilization is suggested, and if selected, neurovascular-sparing clitoroplasty for severe clitoromegaly is suggested (45). With early management (started <2 years of age), 46,XX patients generally have a satisfactory psychosocial outcome (77–79).

In male newborns with severe hypospadias, urological surgery is certainly indicated for functional repair (80). Although a recent review (81) found that 80% of men are satisfied with childhood hypospadias repair, it is advisable to refrain from invasive surgery that is not essential for health and to encourage patient participation and decisions in the choices regarding the sexual sphere (82).

### Psychological Support

Diagnosis of classic CAH during neonatal age activates concerns and anxiety in parents related to the risk of electrolyte crises, genital ambiguity at birth, and the effects of hyperandrogenism on the brain, gender behavior, and body perception. The option of genital surgery, in case of highly virilized genitalia, represents a strong stress factor for families. Although studies in the literature show controversial results regarding the quality of life of people with CAH, case reports show that they can be psychosocial consequences related to the ambiguity of the genitalia (impaired bodily self-image, stigmatization, etc.). Therefore, we believe that psychological support is a useful complement to endocrinological and surgical management, in agreement with the Endocrine Society guidelines (45).

## Author Contributions

AB and FB: conceptualization, methodology, software, CYP11A1-D, CYP21A2-D, and writing—review and editing. AC: resources. AB, FB, and RO: data curation. AB: writing—original draft preparation. RO: StAR-D and CYP17A1-D. AC and VD: 3β-HSD2D and CYP11B1-D. FB, LB, and SM: POR-D. AB and AC: supervision. All authors contributed to the article and approved the submitted version.

## Conflict of Interest

The authors declare that the research was conducted in the absence of any commercial or financial relationships that could be construed as a potential conflict of interest.

## Abbreviations

Δ4A: Δ4-androstenedione
11KΔ4A: 11-keto-androstenedione
11KT: 11-keto-testosterone
17OHP: 17α-hydroxyprogesterone
17OHPreg: 17α-hydroxypregnenolone
Aldo: aldosterone
AMH: anti-Mullerian hormone
Andr: androgens
B: corticosterone
CAH: congenital adrenal hyperplasia
CYP11A1-D: P450 cytochrome side-chain cleavage deficiency
CYP11B1-D: 11β-hydroxylase deficiency
CYP17A1-D: 17α-hydroxylase/17-20 lyase deficiency
CYP21A2-D: 21-hydroxylase deficiency
DHEA: dehydroepiandrosterone
DHT: dihydrotestosterone
DOC: deoxycorticosterone
E1: estrone
E2: estradiol
E3: estriol
F: cortisol
EGS: External Genitalia Score
EMS: External Masculinization Score
GC: glucocorticoid
HDS3B2-D: 3β-hydroxysteroid dehydrogenase deficiency
IMM: inner mitochondrial membrane
LC/MSMS: liquid chromatography/tandem mass spectrometry
MC: mineralocorticoid
NIPD: non-invasive prenatal diagnosis
NSP: newborn screening program
OMM: outer mitochondrial membrane
POR-D: P450 oxidoreductase deficiency
Preg: pregnenolone
Prog: progesterone
S: 11-deoxycortisol
StAR-D: steroidogenic acute regulatory deficiency
SV: simple virilising
SW: salt wasting
T: testosterone
THDOC: tetrahydro-deoxycorticosterone
THS: tetrahydro-deoxycortisol
US: ultrasound.

## References

[1] Baronio F Ortolano R Menabò S Cassio A Baldazzi L Di Natale V. 46,XX DSD due to androgen excess in monogenic disorders of steroidogenesis: genetic, biochemical, and clinical features. Int J Mol Sci. (2019) 20:4605. 10.3390/ijms20184605 31533357 PMC6769793

[2] Baranowski ES Arlt W Idkowiak J. Monogenic disorders of adrenal steroidogenesis. Horm Res Paediatr. (2018) 89:292–310. 10.1159/000488034 29874650 PMC6067656

[3] Yoo H-W Kim G-H. Molecular and clinical characterization of korean patients with congenital lipoid adrenal hyperplasia. J Pediatr Endocrinol Metab. (1998) 11:707–11. 10.1515/JPEM.1998.11.6.707 9829224

[4] Nakae J. Analysis of the steroidogenic acute regulatory protein (StAR) gene in Japanese patients with congenital lipoid adrenal hyperplasia. Hum Mol Genet. (1997) 6:571–6. 10.1093/hmg/6.4.571 9097960

[5] Bose HS Sugawara T Strauss JF Miller WL. The pathophysiology and genetics of congenital lipoid adrenal hyperplasia. N Engl J Med. (1996) 335:1870–9. 10.1056/NEJM199612193352503 8948562

[6] Flück CE Maret A Mallet D Portrat-Doyen S Achermann JC Leheup B. A novel mutation L260P of the steroidogenic acute regulatory protein gene in three unrelated patients of swiss ancestry with congenital lipoid adrenal hyperplasia. J Clin Endocrinol Metab. (2005) 90:5304–8. 10.1210/jc.2005-0874 15985476

[7] Kallali W Gray E Mehdi MZ Lindsay R Metherell LA Buonocore F. Long-term outcome of partial P450 side-chain cleavage enzyme deficiency in three brothers: the importance of early diagnosis. Eur J Endocrinol. (2020) 182:K15–K24. 10.1530/EJE-19-0696 31917682 PMC7087497

[8] Sahakitrungruang T Tee MK Blackett PR Miller WL. Partial defect in the cholesterol side-chain cleavage enzyme P450scc (CYP11A1) resembling nonclassic congenital lipoid adrenal hyperplasia. J Clin Endocrinol Metab. (2011) 96:792–8. 10.1210/jc.2010-1828 21159840 PMC3047228

[9] Chen X Baker BY Abduljabbar MA Miller WL. A Genetic isolate of congenital lipoid adrenal hyperplasia with atypical clinical findings. J Clin Endocrinol Metab. (2005) 90:835–40. 10.1210/jc.2004-1323 15546900

[10] Gassner HL Toppari J Quinteiro González S Miller WL. Near-miss apparent SIDS from adrenal crisis. J Pediatr. (2004) 145:178–83. 10.1016/j.jpeds.2004.04.052 15289763

[11] Miller WL. Disorders in the initial steps of steroid hormone synthesis. J Steroid Biochem Mol Biol. (2017) 165:18–37. 10.1016/j.jsbmb.2016.03.009 26960203

[12] Fujieda K Tajima T Nakae J Sageshima S Tachibana K Suwa S. Spontaneous puberty in 46,XX subjects with congenital lipoid adrenal hyperplasia. Ovarian steroidogenesis is spared to some extent despite inactivating mutations in the steroidogenic acute regulatory protein (StAR) gene. J Clin Invest. (1997) 99:1265–71. 10.1172/JCI119284 9077535 PMC507941

[13] Bose HS Pescovitz OH Miller WL Spontaneous feminization in a 46,XX female patient with congenital lipoid adrenal hyperplasia due to a homozygous frameshift mutation in the steroidogenic acute regulatory protein 1. J Clin Endocrinol Metab. (1997) 82:1511–5. 10.1210/jcem.82.5.3962 9141542

[14] Hauffa B Hiort O. P450 side-chain cleavage deficiency - a rare cause of congenital adrenal hyperplasia. Endocr Dev. (2011) 20:54–62. 10.1159/000321215 21164259

[15] El-Maouche D Arlt W Merke DP. Congenital adrenal hyperplasia. Lancet. (2017) 390:2194–210. 10.1016/S0140-6736(17)31431-9 28576284

[16] Tajima T Fujieda K Kouda N Nakae J Miller WL. Heterozygous mutation in the cholesterol side chain cleavage enzyme (P450scc) gene in a patient with 46,XY sex reversal and adrenal insufficiency. J Clin Endocrinol Metab. (2001) 86:3820–5. 10.1210/jcem.86.8.7748 11502818

[17] Papadimitriou DT Bothou C Zarganis D Karantza M Papadimitriou A. Heterozygous mutations in the cholesterol side-chain cleavage enzyme gene (CYP11A1) can cause transient adrenal insufficiency and life-threatening failure to thrive. Hormones. (2018) 17:419–21. 10.1007/s42000-018-0048-y 29995203

[18] Miller WL Auchus RJ. The molecular biology, biochemistry, and physiology of human steroidogenesis and its disorders. Endocr Rev. (2011) 32:81–151. 10.1210/er.2010-0013 21051590 PMC3365799

[19] Miller WL. Why Nobody Has P450scc (20,22 Desmoslase) deficiency g. J Clin Endocrinol Metab. (1998) 83:1399–400. 10.1210/jcem.83.4.4734-7 9543177

[20] Hiort O Holterhus P-M Werner R Marschke C Hoppe U Partsch C-J. Homozygous disruption of P450 side-chain cleavage (CYP11A1) is associated with prematurity, complete 46,XY sex reversal, and severe adrenal failure. J Clin Endocrinol Metab. (2005) 90:538–41. 10.1210/jc.2004-1059 15507506

[21] Kim CJ Lin L Huang N Quigley CA AvRuskin TW Achermann JC. Severe combined adrenal and gonadal deficiency caused by novel mutations in the cholesterol side chain cleavage enzyme, P450scc. J Clin Endocrinol Metab. (2008) 93:696–702. 10.1210/jc.2007-2330 18182448 PMC2266942

[22] Tee MK Abramsohn M Loewenthal N Harris M Siwach S Kaplinsky A. Varied clinical presentations of seven patients with mutations in CYP11A1 encoding the cholesterol side-chain cleavage enzyme, P450scc. J Clin Endocrinol Metab. (2013) 98:713–20. 10.1210/jc.2012-2828 23337730 PMC3565115

[23] Voutilainen R Miller WL. Developmental expression of genes for the stereoidogenic enzymes P450scc (20,22-Desmolase), P450cl7 (17αHydroxylase/17,20-Lyase), and P450c21 (21-Hydroxylase) in the human fetus*. J Clin Endocrinol Metab. (1986) 63:1145–50. 10.1210/jcem-63-5-1145 3489728

[24] Rubtsov P Karmanov M Sverdlova P Spirin P Tiulpakov A. A novel homozygous mutation in CYP11A1 gene is associated with late-onset adrenal insufficiency and hypospadias in a 46,XY patient. J Clin Endocrinol Metab. (2009) 94:936–9. 10.1210/jc.2008-1118 19116240

[25] Parajes S Chan AO But WM Rose IT Taylor AE Dhir V. Delayed diagnosis of adrenal insufficiency in a patient with severe penoscrotal hypospadias due to two novel P450 side-change cleavage enzyme (CYP11A1) mutations (p.R360W; p.R405X). Eur J Endocrinol. (2012) 167:881–5. 10.1530/EJE-12-0450 22968487 PMC3494866

[26] Abdulhadi-Atwan M Jean A Chung WK Meir K Ben Neriah Z Stratigopoulos G. Role of a founder c.201_202delCT mutation and new phenotypic features of congenital lipoid adrenal hyperplasia in palestinians. J Clin Endocrinol Metab. (2007) 92:4000–8. 10.1210/jc.2007-1306 17666473

[27] Miller WL. MECHANISMS IN ENDOCRINOLOGY: Rare defects in adrenal steroidogenesis. Eur J Endocrinol. (2018) 179:R125–R41. 10.1530/EJE-18-0279 29880708

[28] Al Alawi AM Nordenström A Falhammar H. Clinical perspectives in congenital adrenal hyperplasia due to 3β-hydroxysteroid dehydrogenase type 2 deficiency. Endocrine. (2019) 63:407–21. 10.1007/s12020-018-01835-3 30719691 PMC6420607

[29] Simard J Moisan AM Morel Y Congenital adrenal hyperplasia due to 3beta-hydroxysteroid dehydrogenase/Delta(5)-Delta(4) isomerase deficiency. Semin Reprod Med. (2002) 20:255–76. 10.1055/s-2002-35373 12428206

[30] Guran T Kara C Yildiz M Bitkin EC Haklar G Lin J-C. Revisiting classical 3β-hydroxysteroid dehydrogenase 2 deficiency: lessons from 31 pediatric cases. J Clin Endocrinol Metab. (2020) 105:dgaa022. 10.1210/clinem/dgaa022 31950145

[31] van der Straaten S Springer A Zecic A Hebenstreit D Tonnhofer U Gawlik A. The external genitalia score (EGS): a European multicenter validation study. J Clin Endocrinol Metab. (2019) 105:dgz142. 10.1210/clinem/dgz142 31665438

[32] Ahmed SF Khwaja O Hughes IA. The role of a clinical score in the assessment of ambiguous genitalia. BJU Int. (2000) 85:120–4. 10.1046/j.1464-410x.2000.00354.x 10619959

[33] Prader A. [Perfect male external genital development and salt-loss syndrome in girls with congenital adrenogenital syndrome]. Helv Paediatr Acta. (1958) 13:5–14. 13524803

[34] Benkert AR Young M Robinson D Hendrickson C Lee PA Strauss KA. Severe salt-losing 3β-hydroxysteroid dehydrogenase deficiency: treatment and outcomes of HSD3B2 c.35G>A homozygotes. J Clin Endocrinol Metab. (2015) 100:E1105–E15. 10.1210/jc.2015-2098 26079780

[35] Nordenström A Forest MG Wedell A. A case of 3beta-hydroxysteroid dehydrogenase type II (HSD3B2) deficiency picked up by neonatal screening for 21-hydroxylase deficiency: difficulties and delay in etiologic diagnosis. Horm Res. (2007) 68:204–8. 10.1159/000102593 17496421

[36] Levy-Shraga Y Pinhas-Hamiel O. High 17-hydroxyprogesterone level in newborn screening test for congenital adrenal hyperplasia. BMJ Case Rep. (2016) 2016:bcr2015213939. 10.1136/bcr-2015-213939 26912766 PMC4769481

[37] Araújo VGB de Oliveira RS de Gameleira KPD Cruz CB Lofrano-Porto A. 3β-hydroxysteroid dehydrogenase type II deficiency on newborn screening test. Arq Bras Endocrinol Metabol. (2014) 58:650–5. 10.1590/0004-2730000003098 25211449

[38] Krone N Hughes BA Lavery GG Stewart PM Arlt W Shackleton CHL. Gas chromatography/mass spectrometry (GC/MS) remains a pre-eminent discovery tool in clinical steroid investigations even in the era of fast liquid chromatography tandem mass spectrometry (LC/MS/MS). J Steroid Biochem Mol Biol. (2010) 121:496–504. 10.1016/j.jsbmb.2010.04.010 20417277 PMC2941839

[39] Breder ISS Garmes HM Mazzola TN Maciel-Guerra AT de Mello MP Guerra-Júnior G. Three new Brazilian cases of 17α-hydroxylase deficiency: clinical, molecular, hormonal, and treatment features. J Pediatr Endocrinol Metab. (2018) 31:937–42. 10.1515/jpem-2017-0521 29982238

[40] Kim SM Rhee JH. A case of 17 alpha-hydroxylase deficiency. Clin Exp Reprod Med. (2015) 42:72–6. 10.5653/cerm.2015.42.2.72 26161337 PMC4496435

[41] Chormanski D Muzio MR. C 17 hydroxylase deficiency. in StatPearls. Treasure Island, FL: StatPearls Publishing. Available online at: http://www.ncbi.nlm.nih.gov/books/NBK546644/ (accessed March 28, 2020). 31536251

[42] Merke DP Bornstein SR. Congenital adrenal hyperplasia. Lancet Lond Engl. (2005) 365:2125–36. 10.1016/S0140-6736(05)66736-0 15964450

[43] Hughes IA Houk C Ahmed SF Lee PA LWPES Consensus Group, ESPE Consensus Group. Consensus statement on management of intersex disorders. Arch Dis Child. (2006) 91:554–63. 10.1136/adc.2006.098319 16624884 PMC2082839

[44] Miller WL Merke DP. Tenascin-X, Congenital adrenal hyperplasia, and the CAH-X syndrome. Horm Res Paediatr. (2018) 89:352–61. 10.1159/000481911 29734195 PMC6057477

[45] Speiser PW Arlt W Auchus RJ Baskin LS Conway GS Merke DP. Congenital adrenal hyperplasia due to steroid 21-Hydroxylase deficiency: an endocrine society* clinical practice guideline. J Clin Endocrinol Metab. (2018) 103:4043–88. 10.1210/jc.2018-01865 30272171 PMC6456929

[46] Swart AC Schloms L Storbeck K-H Bloem LM Toit Td Quanson JL. 11β-Hydroxyandrostenedione, the product of androstenedione metabolism in the adrenal, is metabolized in LNCaP cells by 5α-reductase yielding 11β-hydroxy-5α-androstanedione. J Steroid Biochem Mol Biol. (2013) 138:132–42. 10.1016/j.jsbmb.2013.04.010 23685396

[47] O'Shaughnessy PJ Antignac JP Le Bizec B Morvan M-L Svechnikov K Söder O. Alternative (backdoor) androgen production and masculinization in the human fetus. PLOS Biol. (2019) 17:e3000002. 10.1371/journal.pbio.3000002 30763313 PMC6375548

[48] Jones CM Mallappa A Reisch N Nikolaou N Krone N Hughes BA. Modified-release and conventional glucocorticoids and diurnal androgen excretion in congenital adrenal hyperplasia. J Clin Endocrinol Metab. (2017) 102:1797–806. 10.1210/jc.2016-2855 27845856 PMC5470768

[49] Kamrath C Wettstaedt L Boettcher C Hartmann MF Wudy SA. Androgen excess is due to elevated 11-oxygenated androgens in treated children with congenital adrenal hyperplasia. J Steroid Biochem Mol Biol. (2018) 178:221–8. 10.1016/j.jsbmb.2017.12.016 29277706

[50] Bacila I Adaway J Hawley J Mahdi S Krone R Patel L. Measurement of salivary adrenal-specific androgens as biomarkers of therapy control in 21-hydroxylase deficiency. J Clin Endocrinol Metab. (2019) 104:6417–29. 10.1210/jc.2019-00031 31361321

[51] Balsamo A Cacciari E Piazzi S Cassio A Bozza D Pirazzoli P. Congenital adrenal hyperplasia: neonatal mass screening compared with clinical diagnosis only in the Emilia-Romagna region of Italy, 1980-1995. Pediatrics. (1996) 98:362–7. 8784357

[52] Choi R Park H-D Oh HJ Lee K Song J Lee S-Y. Dried blood spot multiplexed steroid profiling using liquid chromatography tandem mass spectrometry in korean neonates. Ann Lab Med. (2019) 39:263. 10.3343/alm.2019.39.3.269 30623618 PMC6340850

[53] Güran T Tezel B Gürbüz F Selver Eklioglu B Hatipoglu N Kara C. Neonatal screening for congenital adrenal hyperplasia in turkey: a pilot study with 38,935 infants. J Clin Res Pediatr Endocrinol. (2019) 11:13–3. 10.4274/jcrpe.galenos.2018.2018.0117 30111524 PMC6398187

[54] New MI Tong YK Yuen T Jiang P Pina C Chan KCA. Noninvasive prenatal diagnosis of congenital adrenal hyperplasia using cell-free fetal DNA in maternal plasma. J Clin Endocrinol Metab. (2014) 99:E1022–E30. 10.1210/jc.2014-1118 24606108 PMC4037720

[55] Chabraoui L Abid F Menassa R Gaouzi A El Hessni A Morel Y Three novel CYP11B1 mutations in congenital adrenal hyperplasia due to steroid 11beta-hydroxylase deficiency in a moroccan population. Horm Res Paediatr. (2010) 74:182–9. 10.1159/000281417 20523022

[56] Zachmann M Tassinari D Prader A. Clinical and biochemical variability of congenital adrenal hyperplasia due to llβ-hydroxylase deficiency, a study of 25 patients*. J Clin Endocrinol Metab. (1983) 56:222–9. 10.1210/jcem-56-2-222 6296182

[57] Menabò S Polat S Baldazzi L Kulle AE Holterhus P-M Grötzinger J. Congenital adrenal hyperplasia due to 11-beta-hydroxylase deficiency: functional consequences of four CYP11B1 mutations. Eur J Hum Genet. (2014) 22:610–6. 10.1038/ejhg.2013.197 24022297 PMC3992560

[58] Holcombe JH Keenan BS Nichols BL Kirkland RT Clayton GW. Neonatal salt loss in the hypertensive form of congenital adrenal hyperplasia. Pediatrics. (1980) 65:777–81. 6966049

[59] Flück CE Tajima T Pandey AV Arlt W Okuhara K Verge CF. Mutant P450 oxidoreductase causes disordered steroidogenesis with and without Antley-Bixler syndrome. Nat Genet. (2004) 36:228–30. 10.1038/ng1300 14758361

[60] Dean B Chrisp GL Quartararo M Maguire AM Hameed S King BR. P450 oxidoreductase deficiency: a systematic review and meta-analysis of genotypes, phenotypes, and their relationships. J Clin Endocrinol Metab. (2020) 105:e42–e52. 10.1210/clinem/dgz255 31825489

[61] Burkhard FZ Parween S Udhane SS Flück CE Pandey AV. P450 Oxidoreductase deficiency: analysis of mutations and polymorphisms. J Steroid Biochem Mol Biol. (2017) 165:38–50. 10.1016/j.jsbmb.2016.04.003 27068427

[62] Fukami M Nishimura G Homma K Nagai T Hanaki K Uematsu A. Cytochrome P450 oxidoreductase deficiency: identification and characterization of biallelic mutations and genotype-phenotype correlations in 35 Japanese patients. J Clin Endocrinol Metab. (2009) 94:1723–31. 10.1210/jc.2008-2816 19258400

[63] Krone N Reisch N Idkowiak J Dhir V Ivison HE Hughes BA. Genotype-phenotype analysis in congenital adrenal hyperplasia due to P450 oxidoreductase deficiency. J Clin Endocrinol Metab. (2012) 97:E257–67. 10.1210/jc.2011-0640 22162478 PMC3380101

[64] Pandey AV Flück CE. NADPH P450 oxidoreductase: Structure, function, and pathology of diseases. Pharmacol Ther. (2013) 138:229–54. 10.1016/j.pharmthera.2013.01.010 23353702

[65] Riddick DS Ding X Wolf CR Porter TD Pandey AV Zhang Q-Y. NADPH-cytochrome P450 oxidoreductase: roles in Physiology, pharmacology, and toxicology. Drug Metab Dispos. (2013) 41:12–23. 10.1124/dmd.112.048991 23086197 PMC3533425

[66] Nicolo C Flück CE Mullis PE Pandey AV. Restoration of mutant cytochrome P450 reductase activity by external flavin. Mol Cell Endocrinol. (2010) 321:245–52. 10.1016/j.mce.2010.02.024 20188793

[67] Flück CE Mullis PE Pandey AV. Reduction in hepatic drug metabolizing CYP3A4 activities caused by P450 oxidoreductase mutations identified in patients with disordered steroid metabolism. Biochem Biophys Res Commun. (2010) 401:149–53. 10.1016/j.bbrc.2010.09.035 20849814

[68] Ono T Bloch K. Solubilization and partial characterization of rat liver squalene epoxidase. J Biol Chem. (1975) 250:1571–9. 234459

[69] Antley R Bixler D. Trapezoidocephaly, midfacial hypoplasia and cartilage abnormalities with multiple synostoses and skeletal fractures. Birth Defects Orig Artic Ser. (1975) 11:397–401. 1227559

[70] Idkowiak J O'Riordan S Reisch N Malunowicz EM Collins F Kerstens MN. Pubertal presentation in seven patients with congenital adrenal hyperplasia due to P450 oxidoreductase deficiency. J Clin Endocrinol Metab. (2011) 96:E453–E62. 10.1210/jc.2010-1607 21190981 PMC3124345

[71] Papadakis GE Dumont A Bouligand J Chasseloup F Raggi A Catteau-Jonard S. Non-classic cytochrome P450 oxidoreductase deficiency strongly linked with menstrual cycle disorders and female infertility as primary manifestations. Hum Reprod. (2020) 35:939–49. 10.1093/humrep/deaa020 32242900

[72] Bonamichi BDSF Santiago SLM Bertola DR Kim CA Alonso N Mendonca BB. Long-term follow-up of a female with congenital adrenal hyperplasia due to P450-oxidoreductase deficiency. Arch Endocrinol Metab. (2016) 60:500–4. 10.1590/2359-3997000000213 27737328 PMC10118638

[73] Shackleton C Marcos J Arlt W Hauffa BP. Prenatal diagnosis of P450 oxidoreductase deficiency (ORD): a disorder causing low pregnancy estriol, maternal and fetal virilization, and the Antley-Bixler syndrome phenotype. Am J Med Genet. (2004) 129A:105–12. 10.1002/ajmg.a.30171 15316970

[74] Xu D Lu L Xi L Cheng R Pei Z Bi Y. Efficacy and safety of percutaneous administration of dihydrotestosterone in children of different genetic backgrounds with micropenis. J Pediatr Endocrinol Metab. (2017) 30:1285–91. 10.1515/jpem-2016-0400 29176021

[75] Geley S Kapelari K Jöhrer K Peter M Glatzl J Vierhapper H. CYP11B1 mutations causing congenital adrenal hyperplasia due to 11 beta-hydroxylase deficiency. J Clin Endocrinol Metab. (1996) 81:2896–901. 10.1210/jcem.81.8.8768848 8768848

[76] Inauen C Rüfenacht V Pandey AV Hu L Blom H Nuoffer J-M. Effect of cysteamine on mutant ASL proteins with cysteine for arginine substitutions. Mol Diagn Ther. (2016) 20:125–33. 10.1007/s40291-015-0182-z 26745957

[77] Suorsa KI Mullins AJ Tackett AP Scott Reyes KJ Austin P Baskin L. Characterizing early psychosocial functioning of parents of children with moderate to severe genital ambiguity due to disorders of sex development. J Urol. (2015) 194:1737–42. 10.1016/j.juro.2015.06.104 26196734 PMC5889973

[78] Almasri J Zaiem F Rodriguez-Gutierrez R Tamhane SU Iqbal AM Prokop LJ. Genital reconstructive surgery in females with congenital adrenal hyperplasia: a systematic review and meta-analysis. J Clin Endocrinol Metab. (2018) 103:4089–96. 10.1210/jc.2018-01863 30272250

[79] Razzaghy-Azar M Karimi S Shirazi E. Gender identity in patients with congenital adrenal hyperplasia. Int J Endocrinol Metab. (2017) 15:e12537. 10.5812/ijem.12537 29201068 PMC5701969

[80] Snodgrass W Bush N. Is distal hypospadias repair mostly a cosmetic operation? J Pediatr Urol. (2018) 14:339–40. 10.1016/j.jpurol.2018.06.004 29970334

[81] Tack LJW Springer A Riedl S Tonnhofer U Weninger J Hiess M. Psychosexual outcome, sexual function, and long-term satisfaction of adolescent and young adult men after childhood hypospadias repair. J Sex Med. (2020) 17:1665–75. 10.1016/j.jsxm.2020.04.002 32444342

[82] Roen K Hegarty P. Shaping parents, shaping penises: How medical teams frame parents' decisions in response to hypospadias. Br J Health Psychol. (2018) 23:967–81. 10.1111/bjhp.12333 30054962

